# Bench to Bedside Development of [^18^F]Fluoromethyl-(1,2-^2^H_4_)choline ([^18^F]D4-FCH)

**DOI:** 10.3390/molecules28248018

**Published:** 2023-12-08

**Authors:** Amarnath Challapalli, Tara D. Barwick, Suraiya R. Dubash, Marianna Inglese, Matthew Grech-Sollars, Kasia Kozlowski, Henry Tam, Neva H. Patel, Mathias Winkler, Penny Flohr, Azeem Saleem, Amit Bahl, Alison Falconer, Johann S. De Bono, Eric O. Aboagye, Stephen Mangar

**Affiliations:** 1 Department of Surgery and Cancer, Imperial College London, Hammersmith Hospital Campus, Du Cane Road, London W12 0NN, UK; amarnath.challapalli@uhbw.nhs.uk (A.C.); tara.barwick@nhs.net (T.D.B.); suraiya.dubash@nhs.net (S.R.D.); marianna.inglese17@imperial.ac.uk (M.I.); m.grech-sollars@ucl.ac.uk (M.G.-S.); kaskoz@yahoo.com (K.K.); 2 Department of Clinical Oncology, Bristol Haematology and Oncology Center, Horfield Road, Bristol BS2 8ED, UK; amit.bahl@uhbw.nhs.uk; 3 Department of Radiology & Nuclear Medicine, Imperial College Healthcare NHS Trust, Hammersmith Hospital, Du Cane Road, London W12 0HS, UK; henry.tam@nhs.net (H.T.); neva.patel1@nhs.net (N.H.P.); 4 Department of Urology, Imperial College Healthcare NHS Trust, Charing Cross Hospital, London W6 8RF, UK; mathias.winkler@nhs.net (M.W.); alison.falconer1@nhs.net (A.F.); 5 Division of Clinical Studies, The Institute of Cancer Research and Royal Marsden Hospital, Cotswold Road, Sutton SM2 5NG, UK; penny.flohr@icr.ac.uk (P.F.); johann.debono@icr.ac.uk (J.S.D.B.); 6 Invicro, A Konica Minolta Company, Burlington Danes Building, Hammersmith Hospital, Du Cane Road, London W12 0NN, UK; azeem.saleem@hyms.ac.uk; 7 Hull York Medical School, University of Hull, Cottingham Road, Hull HU6 7RX, UK

**Keywords:** [^18^F]Fluoromethyl-(1,2-^2^H_4_)choline, positron emission tomography, choline, cell membrane metabolism, in vivo, in vitro, dosimetry, metastatic castrate-resistant prostate cancer

## Abstract

Malignant transformation is characterised by aberrant phospholipid metabolism of cancers, associated with the upregulation of choline kinase alpha (CHKα). Due to the metabolic instability of choline radiotracers and the increasing use of late-imaging protocols, we developed a more stable choline radiotracer, [^18^F]fluoromethyl-[1,2-^2^H_4_]choline ([^18^F]D4-FCH). [^18^F]D4-FCH has improved protection against choline oxidase, the key choline catabolic enzyme, via a ^1^H/^2^D isotope effect, together with fluorine substitution. Due to the promising mechanistic and safety profiles of [^18^F]D4-FCH in vitro and preclinically, the radiotracer has transitioned to clinical development. [^18^F]D4-FCH is a safe positron emission tomography (PET) tracer, with a favourable radiation dosimetry profile for clinical imaging. [^18^F]D4-FCH PET/CT in lung and prostate cancers has shown highly heterogeneous intratumoral distribution and large lesion variability. Treatment with abiraterone or enzalutamide in metastatic castrate-resistant prostate cancer patients elicited mixed responses on PET at 12–16 weeks despite predominantly stable radiological appearances. The sum of the weighted tumour-to-background ratios (TBRs-wsum) was associated with the duration of survival.

## 1. Introduction

Choline is one of the components of phosphatidylcholine (PC), an essential element of phospholipids in the cell membrane [1] and is required for structural stability and cell proliferation. Tumours show a high proliferation and increased cell membrane components that will lead to an increased uptake of choline. The progression of normal cells to malignant phenotypes is associated with altered membrane choline phospholipid metabolism [2].

Choline kinaseα (CHKα), the first enzyme in the Kennedy pathway [3], is responsible for the de novo synthesis of PC and the generation of phosphorylcholine (PCho) from its precursor, choline. CHKα has been extensively linked to cell proliferation and human carcinogenesis [4,5,6]. A strong correlation between CHKα activity and cancer onset has been established by providing evidence that CHK dysregulation is a frequent event occurring in a variety of human tumours, such as breast, lung, colorectal, and prostate tumours [6,7]. Therefore, choline kinase activity represents a potential biomarker for diagnostic use in oncology [8,9]. The use of positron emission tomography (PET) to measure choline transport and CHKα activity is the subject of this review article.

Nature-identical radiolabelled choline represented the first probe to be established for measuring choline tumour uptake by using PET; the main biological variable of interest being the maximum voxel Standardised Uptake Value (SUV_max_). Hara et al. in the first report of the use of [^11^C]choline in prostate cancer, noted that the SUV_max_ of prostate cancer was three-fold greater than that of normal prostate tissue or of benign prostatic hyperplasia (BPH) [10]. Although [^11^C]choline injection is approved for use in men with recurrent prostate cancer by the US Food and Drug Administration, the short half-life of carbon-11 (t_½_ = 20.1 min) limits the application of [^11^C]choline to centres with an on-site cyclotron. As a result, fluorine-18 (t_½_ = 109.8 min)-labelled choline analogues were independently developed by Hara et al. [11] ([^18^F]fluoroethylcholine (FEC)) and by DeGrado et al. [12] ([^18^F]fluoromethylcholine (FMC)). In theory, the longer half-life of [^18^F] (109.8 min) is potentially advantageous in permitting the late imaging of tumours when sufficient clearance of the parent tracer in systemic circulation has occurred.

## 2. Deuterated Choline

**Figure 1:**
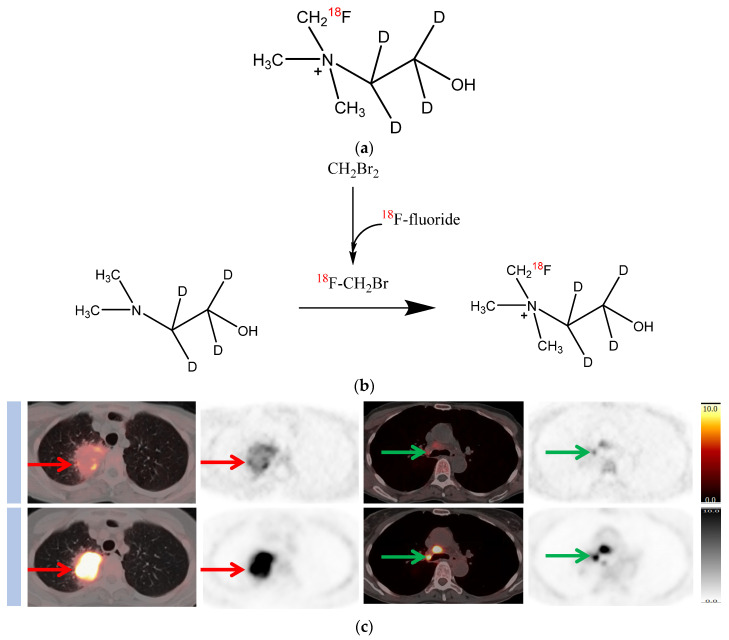
Chemical structure and synthesis of [^18^F]D4-FCH, and images of [^18^F]D4-FCH-PET in patients with non-small cell lung cancer (NSCLC). (**a**) Chemical structure of [^18^F]D4-FCH. (**b**) Synthesis of [^18^F]D4-FCH. No-carrier-added [^18^F]D4-FCH is synthesised by reacting [^18^F]fluorobromomethane, synthesised from dibromomethane by [^18^F]fluoride-bromide substitution, with D4-*N*,*N*-dimethylaminoethanol precursor. (**c**) [^18^F]D4-FCH (top row) and [^18^F]FDG PET/CT images (bottom row) in a patient with NSCLC right upper lobe primary (red arrows) demonstrating tumour heterogeneity, and right paratracheal lymph node (green arrows).

[^11^C]Choline and [^18^F]FMC are readily oxidized by choline oxidase mainly in kidney and liver tissues, with metabolites detectable in the plasma soon after injection of the radiotracer [13,14,15]. This causes difficulty in highlighting the relative contributions of parent radiotracers and catabolites to the PET signal when a late-imaging protocol is used. A more metabolically stable fluorocholine analogue, [^18^F]fluoromethyl-[1,2-^2^H_4_]choline ([^18^F]D4-FCH), has been developed based on the deuterium isotope effect [16]. The simple substitution of deuterium (^2^D) for hydrogen (^1^H) and the presence of ^18^F reduces the degradation of the parent tracer and improves stability [15,17,18]. This modification is hypothesised to increase the net availability of the parent tracer for phosphorylation and intracellular trapping leading to a better signal-to-background contrast, thus improving sensitivity. The translational studies of [^18^F]D4-FCH from bench to bedside are summarised in Table 1. In this article, we elaborate the development of the more stable choline radiotracer, [^18^F]D4-FCH (Figure 1a; [^18^F]D4-FCH has improved protection against choline oxidase, the key choline catabolic enzyme, via a ^1^H/^2^D isotope effect, together with fluorine substitution). We discuss the steps in the translation of the new radiotracer from in vitro discovery to preclinical imaging, in first in-human studies, in an evaluation in lung cancer patients, and then finally in an evaluation of its role in response assessment in metastatic prostate cancer patients. The initial part of the paper reviews the various preclinical and clinical studies of [^18^F]D4-FCH. In the section on response assessment, we discuss our findings regarding the role of [^18^F]D4-FCH in the response assessment to novel antiandrogens in metastatic prostate cancer patients.

## 3. Synthesis and Stability

[^18^F]D4-FCH was prepared in a two-step reaction with a 12% overall yield, a final radiochemical purity of >99%, and a total synthesis time of 150 min. Alkylation with [^18^F]fluoromethyl tosylate proved to be the most reliable radiosynthetic route [15].

The effect of deuterium substitution on bond strength was tested by evaluation of the chemical oxidation pattern using potassium permanganate. Chromatographic analyses showed that [^18^F]D4-FCH was ~80% intact after treatment for 1 h with potassium permanganate, which oxidizes choline to choline betaine. Conversely, only 40% of nondeuterated FCH was intact. In the presence of choline oxidase, [^18^F]D4-FCH was 29 ± 4% intact after treatment for 1 h, whereas only 11 ± 8% of FCH was intact. Therefore, [^18^F]D4-FCH is more stable than FCH against oxidation [15].

## 4. In Vivo Biodistribution

In a murine HCT116 human colon xenograft model, in vivo biodistribution patterns of [^18^F]D4-FCH, and [^18^F]FCH were compared at 2, 30, and 60 min after injection [15]. [^18^F]D4-FCH exhibited a higher accumulation in the tumours than [^18^F]FCH. The distribution of all FCH radiotracers showed a similar uptake profile in most organs, with prominent radioactivity in the kidneys and liver. A pronounced increase in tumour uptake of [^18^F]D4-FCH at the later time points was evident. A reduced uptake of [^18^F]D4-FCH, relative to [^18^F]FCH, in lung tissue may make imaging of thoracic tumours using this radiotracer superior to [^18^F]FCH.

## 5. In Vivo Metabolic Stability

[^18^F]D4-FCH was validated for imaging tumours preclinically and was found to be a very promising, metabolically stable radiotracer for imaging choline metabolism in tumours [17,18]. The improved stability (protection against oxidation by choline oxidase) is conferred by a ^1^H/^2^D isotope effect, together with fluorine substitution [17,18].

## 6. In Vivo Response Assessment

The use of [^18^F]D4-FCH for imaging response to CHK inhibition represents the most direct biomarker investigation. Trousil et al. showed that [^18^F]D4-FCH reports reduction in phosphocholine formation when mice are treated with the CHK inhibitor ICL-CCIC-0019 [22]. Furthermore, Leyton et al. have demonstrated a marked reduction in radiotracer retention (all imaging variables) in the tumours treated with a mitogenic extracellular kinase inhibitor, despite a reduction in tumour size by only 12% after 10 days of treatment [17]. This suggests that [^18^F]D4-FCH can be used to detect treatment response early during the course of therapy prior to any potential tumour size reduction. More recent studies highlight the role of hypoxia in [^18^F]D4-FCH tumour biomarker response, suggesting that HIF-1α-responsive efflux transporters, including ABCB4, can modulate [^18^F]D4-FCH uptake; consideration of efflux is supported in a mathematical analysis of [^18^F]D4-FCH PET data [20]. Incidentally, an RNA-Seq analysis has identified an upregulation of ABCB1 and ABCB4 transporters as the main mechanisms involved in therapy resistance to CHK inhibitors in pancreatic cancer [23].

## 7. In Vitro and In Vivo Comparison of [^11^C] and [^18^F]choline Analogues

Witney et al. carried out biodistribution, metabolism, small-animal PET studies, and kinetic analysis of [^11^C]choline, [^11^C]methyl-[1,2-_2_H_4_]-choline ([^11^C]D4-choline), and [^18^F]D4-FCH uptake in human colon (HCT116), melanoma (A375), and prostate cancer (PC3-M) xenograft-bearing mice [18]. All tracers were converted intracellularly to their respective phosphocholine analogues. Their analyses have confirmed that deuteration and fluorination combine to provide protection against choline oxidation in vivo. Uptake of [^18^F]D4-FCH was quantitatively similar in three tumours—HCT116, A375, and PC3-M—with similar radiotracer delivery (K_1_) and CHKα expression, suggesting that [^18^F]D4-FCH may have utility for cancer detection irrespective of histologic type.

## 8. Healthy Volunteer Biodistribution

In order to extend the pharmacokinetic aspects of the preclinical findings into human application, a first in-human study of [^18^F]D4-FCH was performed on eight healthy volunteers to evaluate dosimetry, biodistribution, and safety [19]. [^18^F]D4-FCH was well tolerated in all subjects, with no radiotracer-related serious adverse events reported. No significant changes in vital signs, clinical laboratory blood tests, or electrocardiograms were observed. The mean effective dose averaged over both males and females (±SD) was estimated to be 0.025 ± 0.004 (male 0.022 ± 0.002; female 0.027 ± 0.002) mSv/MBq, which is comparable with the ED of [^18^F]FDG (0.019 mSv/MBq) [24]. The five organs receiving the highest-absorbed dose (mGy/MBq) were the kidneys (0.106 ± 0.03), liver (0.094 ± 0.03), pancreas (0.066 ± 0.01), urinary bladder wall (0.047 ± 0.02), and adrenals (0.046 ± 0.01). Elimination was through the renal and hepatic systems. The human biodistribution study has concluded that [^18^F]D4-FCH is a safe PET radiotracer with a dosimetry profile comparable to other common [^18^F] PET tracers.

## 9. First In-Patient (Lung Cancer) Evaluation

Given the low background uptake of [^18^F]D4-FCH in the thorax [19], [^18^F]D4-FCH was studied in 17 newly diagnosed non-small cell lung cancer (NSCLC) patients to evaluate tumour heterogeneity [20]. PET/CT scans were acquired concurrently with radioactive blood sampling to permit mathematical modelling of the blood-tissue transcellular rate constants. Comparisons were made with diagnostic [^18^F]fluorodeoxyglucose (FDG) scans. The [^18^F]D4-FCH-derived uptake (SUV_max_) in index primary lesions (*n* = 17) ranged between 2.87 and 10.13, which was lower than that of [^18^F]FDG-PET (6.89 and 22.64, respectively). Mathematical modelling demonstrated the net irreversible uptake of [^18^F]D4-FCH at steady-state, and parametric mapping of the entire tumour showed large intratumoural heterogeneity in radiotracer retention (Figure 1b). This highlights the potential for radiotherapy dose escalation.

As aforementioned, hypoxia is known to regulate CHKα activity and choline transport through hypoxia-inducible factor-1α (HIF-1α). This may confound the uptake of [^18^F]D4-FCH. Moreover, in dynamic PET scans, most tumours exhibit a rapid radiotracer uptake phase followed by a plateaued time versus radioactivity (TAC) curve, regardless of their oxidative stability [15,18]. This suggests that other mechanisms are at play in tumour-radiolabelled choline biodistribution dynamics [20]. Li et al.’s work relating to the effect of hypoxia on [^18^F]D4-FCH uptake [21] highlighted the export of phosphorylated [^18^F]D4-FCH and [^18^F]D4-FCHP via HIF-1α-responsive efflux transporters, including ABCB4. These findings are supported by the graphical analysis of NSCLC patient data with a compartmental modelling that accounts for the efflux [20].

## 10. Longitudinal Case Study in a Brain Tumour Patient

**Figure 2:**
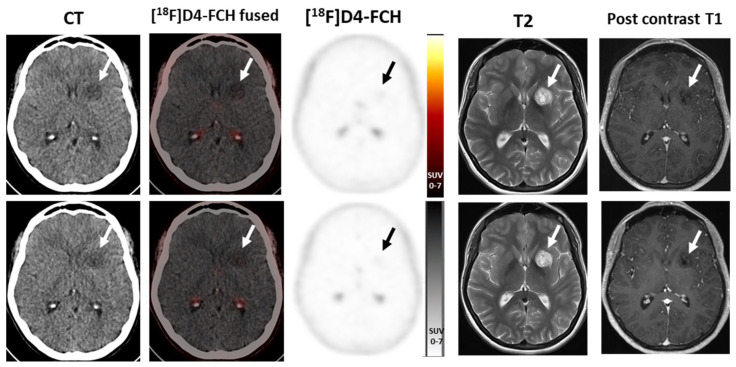
[^18^F]D4–FCH PET/CT in a left thalamic low-grade glioma 6 months apart (**top row**, baseline; **bottom row**, 6 months later) showing minimal tracer uptake above background in the lesion, stable between scans, high signal on T2–weighted MRI imaging with no significant enhancement post-contrast. (Note physiological activity in the choroid plexus). Stable on continued follow-up 6 years later (not shown).

[^18^F]FMC has been previously used to evaluate choline metabolism in patients with primary brain tumours, with one study showing that a combination of [^18^F]FMC uptake on PET/CT and MR spectroscopy correlated with the tumour grade [25]. In order to assess the utility of choline uptake in the prediction of high-grade transformation of low-grade gliomas, a longitudinal case study was carried out using [^18^F]D4-FCH. A patient with a low-grade glioma was recruited to the study following ethical approval and consent. The patient had two PET and MRI scans, 6 months apart. At baseline, a faint tracer uptake was visible in the lesion (Figure 2), which was stable on imaging 6 months later. There has been no history of transformation on routine follow-up.

## 11. Response Evaluation in Metastatic Castrate-Resistant Prostate Cancer (mCRPC): Proof of Concept Study

Limitations of RECIST 1.1 [26], particularly in mCRPC patients with bone metastases [27], has led to the investigation of metabolic PET imaging methods. Other studies using [^18^F]FCH have demonstrated the potential of choline tracers to monitor treatment to abiraterone or enzalutamide at early (3–6 weeks) times post-treatment [28,29]. In this original research, we examined temporal variations of [^18^F]D4-FCH in mCRPC patients on abiraterone or enzalutamide in a prospective nonrandomized feasibility study.

mCRPC patients due to receive abiraterone (1000 mg with twice-daily 5 mg prednisolone in 28-day cycles)/enzalutamide (daily at 160 mg) were enrolled. PET/CT analyses were conducted at baseline, 4–6 weeks, and 12–16 weeks (detailed methodology in Supplementary Table S1). In brief, index lesions ≥10 mm with increased tracer uptake above background structures were outlined. For the follow-up visits, the same index lesions were outlined. For lesions no longer visible above the background, the background activity at the site of the lesion was outlined. Changes in the semiquantitative Standardized Uptake Value (SUV), both SUV_max_ and SUV_mean_, were calculated. In addition, for index lesions, tumour-to-background ratios (TBRs) were documented (SUV_max_ lesion/SUV_mean_ background) using background muscle for nodes and soft-tissue lesions and background bone for bone metastases, for both the early 4–6 week post-therapy scan and the 12–16 week post-therapy scan. For follow-up data, per-patient and per-lesion analyses were performed. [^18^F]D4-FCH PET changes were compared to prostate-specific antigen (PSA) levels, prostate cancer working group 3 (PCWG3) response criteria [30], and survival duration. All statistical tests were run in Matlab (Mathworks, R2018b).

### Patient Characteristics and Optimal Imaging Time for [^18^F]D4-FCH PET in mCRPC

**Figure 3:**
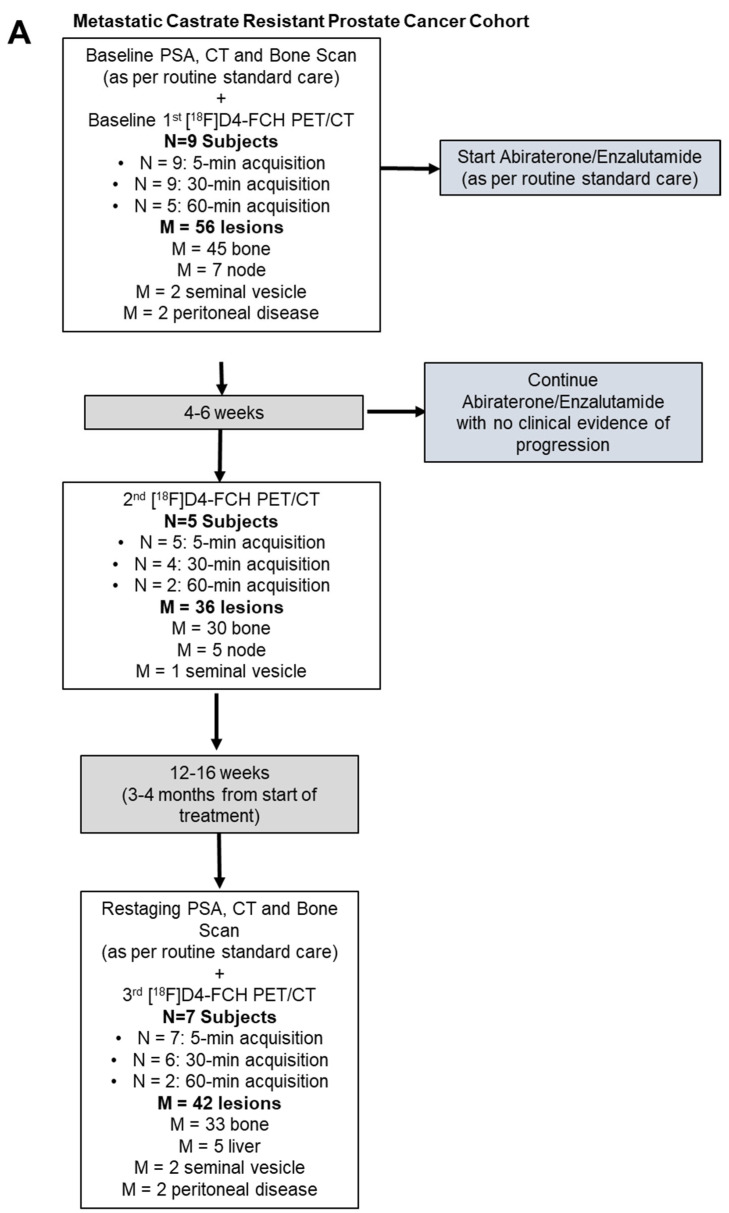

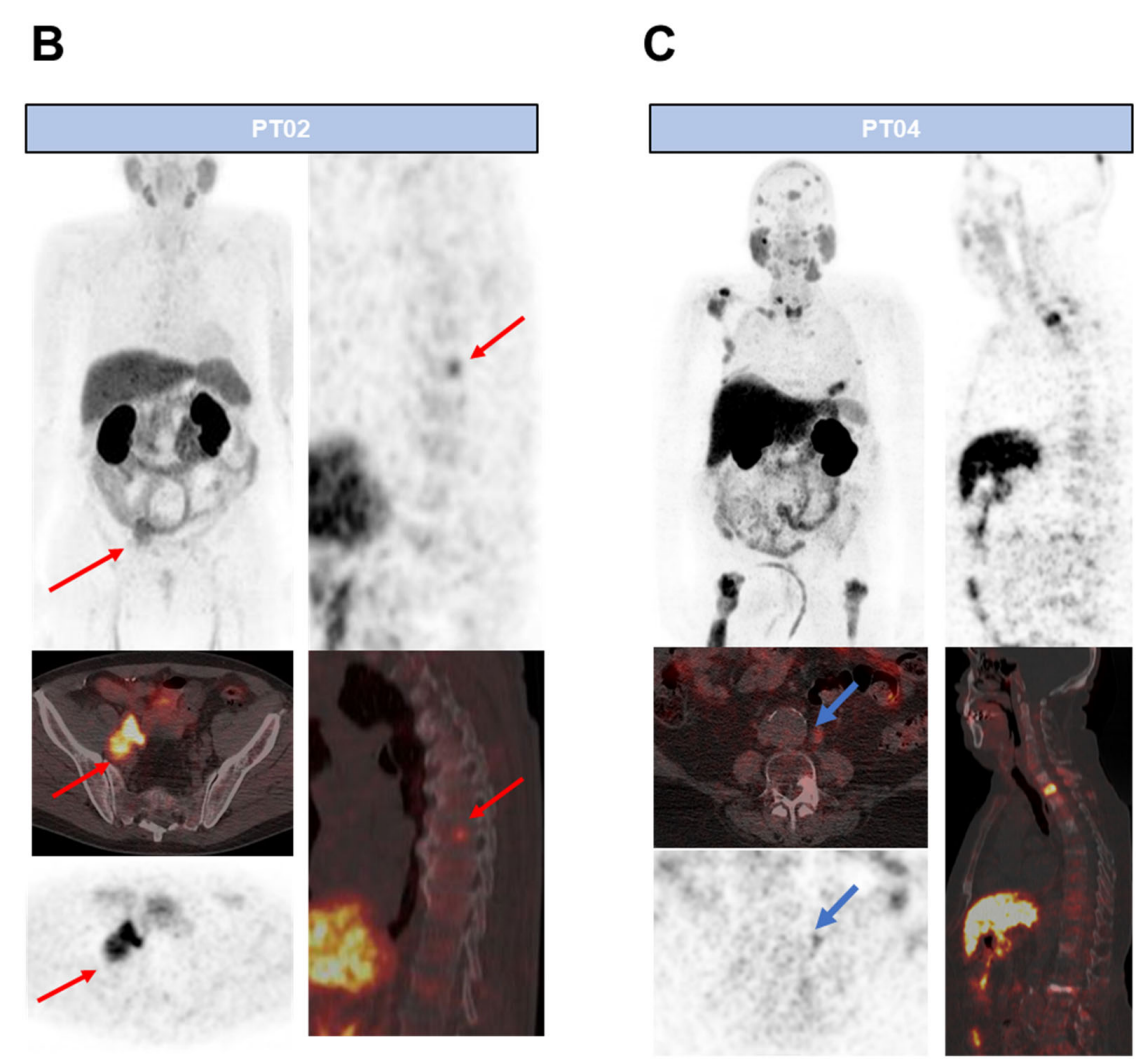
Study design and PET images. (**A**) Details of the PET study and summary of patients and lesions analysed. (**B**) Typical [^18^F]D4-FCH uptake. MIP, axial, and sagittal views of PET and PET/CT of PT02, showing right external iliac node and T8 bone metastasis (red arrows). (**C**) Typical [^18^F]D4-FCH uptake. MIP, axial, and sagittal views of PET and PET/CT of PT04, showing multiple bone metastases and a left paraaortic nodal metastasis (blue arrows).

In this ‘no-benefit trial’, nine patients underwent a baseline PSA measurement, conventional staging, and [^18^F]D4-FCH scan (Table 2). Of these, five patients had an early post-treatment scan at 4–6 weeks post-therapy (median 5.5 weeks; range 3.9–6.4) and seven patients completed midtreatment time points (12–16 weeks) post-therapy (median 15.3 weeks; range 13.1–17.3) (Figure 3). Six patients had stable disease, and one was a partial responder. A total of 55 lesions were documented. Of these, only 8 out of the 55 were measurable by RECIST 1.1 (two peritoneal, five nodes, and one bone, which had a soft-tissue component). Imaging features of mCRPC demonstrate significant response heterogeneity within and between patients (per-lesion analyses).

**Figure 4:**
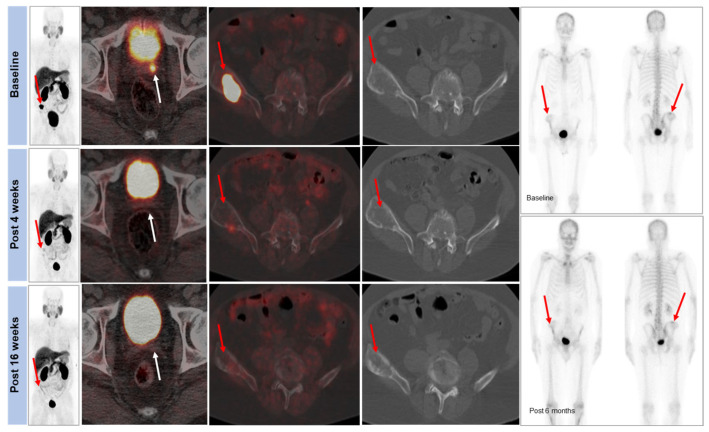
Response heterogeneity—changes in activity of lesions within individual patients at different times—detected by using [^18^F]D4-FCH PET/CT. Representative PET images acquired at baseline (t1), early post-treatment (4–6 weeks; t2), and midtherapy (14–16 weeks; t3) demonstrating decreasing radiotracer uptake in a left seminal vesicle lesion (white arrows) and right iliac bone metastasis (red arrows).

**Figure 5:**
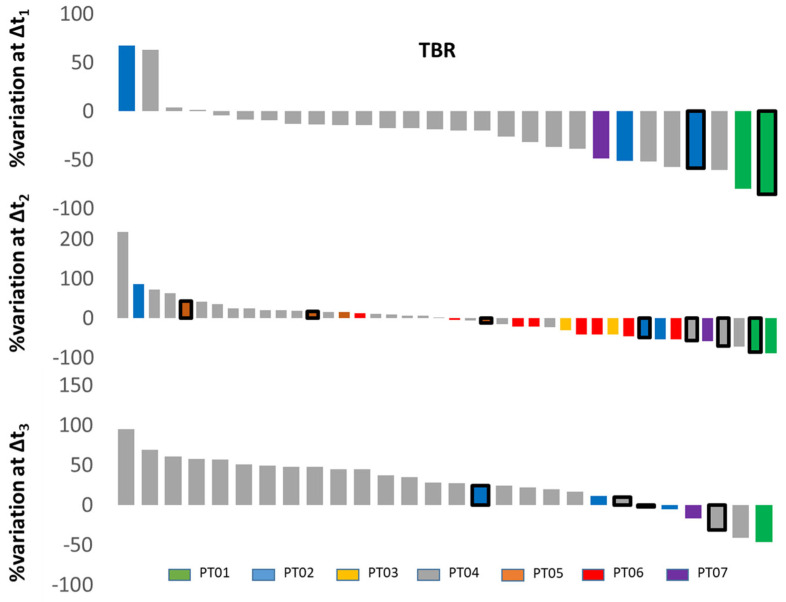
Relative lesional radiotracer uptake at the different time points represented as ‘waterfall’ plots of the percentage variation (%Δ1/2/3) of the tumour-to-background ratio (TBR). We only included patients who completed at least visit 1 and 3 scans within this analysis to avoid bias. The variations were evaluated as follows: %Δt1 = ((t2 − t1)/t1) × 100, n = 28 lesions; %Δt2 = ((t3 − t1)/t1) × 100, n = 48 lesions; %Δt3 = ((t3 − t2)/t2) × 100), n = 28 lesions. TBRBoneMet = SUV_max_BoneMet/SUV_mean_BackgBone. TBRSoftTissueMet = SUV_max_SoftTissueMet/SUV_mean_BackgMuscle (Backg: background). Soft-tissue metastases are identified with black-outlined bars (rest are bone metastases). Subject P02 is classified as partial responder based on PCWG3.

**Figure 6:**
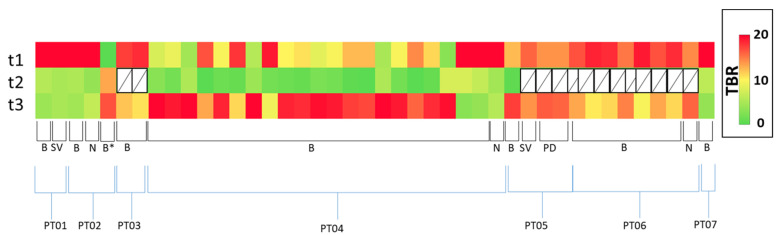
Response plots of the absolute TBR (per-lesion analysis) evaluated at the three time points, represented as ‘heatmaps’. Of the seven patients who had both t1 and t3 scans, those who did not have t2 scans were excluded from the analysis. N: Node; SV: Seminal vesicle; PD: Peritoneal Disease; B: Bone. *: progressive bone metastasis in patient #2.

We found substantial temporal response heterogeneity in patients having either abiraterone or enzalutamide treatment. Significant reduction in [^18^F]D4-FCH uptake on PET was seen in lesions, which were stable on conventional imaging (Figure 4). An initial decrease in TBR was seen in most bone lesions and soft-tissue lesions at visit 2, with subsequent increases in TBR in most lesions by visit 3. This could represent a ‘flare effect’ in the bone lesions. Another pattern was a mixed response (Figure 5). The heatmaps in Figure 6 show that the absolute uptake variable, as opposed to % change, was moderate (at TBR of 6.7) at visit 3 in patient (#2) who recorded a partial response; the bone lesion in this patient recorded a low uptake (TBR of 2.4) at visit 3. Notably, patients # 2, 4, and 5 all had one or more individual lesions with TBR values > 5 at visit 3 (Figure 6) signifying an escape from therapeutic pressures. The aggregate lesion uptake correlates with baseline PSA and associates with survival (per-patient analyses).

TBR-wsum ensured that changes in lesions with low and high cholinic phenotypes alike were adequately captured. Baseline PSA or PSA at different time points was not associated with TBR-wsum variables (Supplementary Figure S1A,B). In contrast, change in PSA at 3 months compared to baseline correlated with similar changes in TBR-wsum (Supplementary Figure S1C). Generally, patients with larger TBR-wsum changes survived longer, with the exception being patient# 2 who had one lesion that showed TBR >5 at visit 3 (Figure 6); this was not the case for PSA or PCWG3 (Supplementary Figure S2A–D). With the exception of patient# 2, TBR-wsum >30% gave a PFS advantage of >25 months, while <30% gives a PFS of <14 months (Supplementary Figure S2A–D). We did not have patients with PD or CR at 3 m post-therapy initiation; hence, the interpretation of these results should be restricted to patients with SD and PR. Neither baseline PSA nor TBR-wsum predicted survival (*p* > 0.05).

In this first use of [^18^F]D4-FCH PET/CT in patients with prostate cancer, we show large interlesion heterogeneity, together with temporal heterogeneity in mCRPC patients undergoing treatment with abiraterone or enzalutamide. The treatment elicited mixed responses on PET at 12–16 weeks despite predominantly stable radiological appearances. We identify lesions that have escaped the selective pressures of cancer therapy and show that aggregate measures of [^18^F]D4-FCH PET/CT from multiple lesions correlate with PSA and are associated with survival, thus providing a quantitative visual reflection of response heterogeneity.

## 12. Discussion

Malignant transformation is characterised by aberrant phospholipid metabolism of cancers [6], associated with an upregulation of CHKα [2]. Choline metabolism has been studied utilizing magnetic resonance spectroscopy (MRS) of tumour tissue biopsies, as well as noninvasive tissue measurement [31]. PET-labelled choline tracers provide improved sensitivity when compared to MRS and enable dynamic measurements of the early steps of choline metabolism. Due to the metabolic instability of choline radiotracers and the increasing use of late-imaging protocols (~60 min, to permit elimination of nonspecific metabolites), we developed a more stable choline radiotracer, [^18^F]D4-FCH [15]. Preclinical studies (Table 1) showed that [^18^F]D4-FCH has improved protection against choline oxidase, the key choline catabolic enzyme, via a ^1^H/^2^D isotope effect, together with fluorine substitution [15,17,18].

To date, [^11^C]choline and [^18^F]FCH have been extensively used for the clinical imaging of prostate, brain, breast, hepatocellular (HCC), and esophageal carcinomas [32,33]. Choline metabolism is altered in gliomas, and pilot clinical studies have shown a differential uptake of choline radiotracers between glioma and normal brain tissue and between gliomas and other disease processes. Sollars et al. have shown that [^18^F]FMC PET/CT differentiated WHO (World Health Organization) grade IV from grade II and III tumours. Tumoural [^18^F]FMC PET-CT uptake was higher than in normal-appearing white matter across all grades and markedly elevated within regions of contrast enhancement. This uptake was independent of choline kinase expression [25].

Tumour differentiation is a major predictive factor of post-operative recurrence in HCC [34]. However, the histological analysis of tumour differentiation, which remains the gold standard, is currently carried out only in atypical cases. Conventional imaging is essential for the management of HCC patients [35], but its limited value motivated the use of PET imaging although still not consensually recommended. As [^18^F]FDG shows limited sensitivity to detect HCC, choline PET has been proposed as a complementary diagnostic tool [36]. The [^18^F]FDG/[^18^F]choline dual-tracer PET behaviour of uptake shows a high overlap between well- and less-differentiated HCC, making the characterization of tumours challenging based on such a PET combination [9]. Using [^18^F]D4-FCH may potentially improve the sensitivity and is worth further evaluation.

We have shown previously that the choline-based radiotracer, [^11^C]choline, which correlates with CHKα expression and represents a proliferation-independent phenotype in prostate cancer [37], decreased predictably following androgen deprivation [38], interpreted as a reduction in choline transport/metabolism or loss of cell viability (similar directionality of change) [39].

Recently, prostate-specific membrane antigen (PSMA)-PET has taken centre stage in functional imaging of prostate cancer and is superseding choline PET. A recent meta-analysis of a head-to-head comparison of detection rates (DRs) between radiolabelled choline and PSMA PET/CT has shown that the overall DR of radiolabelled PSMA PET/CT on a per-patient-based analysis is higher compared to that of radiolabelled choline PET/CT (78% versus 56%, respectively) in the setting of biochemical relapse [40]. This is due to the higher contrast and tumour uptake of PSMA than radiolabelled choline.

In rationalising the use of choline and PSMA PET, it is appreciated that PSMA expression is inversely related to androgen response; androgen deprivation, or abiraterone treatment of human castration-resistant PCa cell line VCaP-stimulated PSMA expression and increased PSMA-based radiotracer uptake [41]. On the other hand, a loss of cell viability will be expected to decrease radiotracer uptake; thus, while human studies with PSMA-PET to monitor therapy response following arbiraterone or enzalutamide have been reported [37,42], the interpretation of the data is challenging [38].

[^18^F]D4-FCH PET/CT shows large lesion variability in response to abiraterone or enzalutamide, suggesting an escape from selective pressures of therapy. PET variables including TBR-wsum predicted the duration of survival, depending on individual lesion response. Future studies should elaborate how this variable, together with progression (escape of the resistant lesion(s)) on PET influences progression-free and overall survival in a larger patient cohort and encourages exploration of choline-transport-targeted theranostics as recently suggested [43].

Choline PET/CT imaging is gathering momentum in the localisation of parathyroid adenomas to guide parathyroid surgery with high detection rates in patients with primary hyperparathyroidism [44]. However, in the more challenging persistent/recurrent primary hyperparathyroidism cases, detection rates are lower and the potential improved sensitivity of [^18^F]D4-FCH PET could be explored in this setting.

Paired [^18^F]FCH and [^18^F]FDG PET have been shown to predict a 6-month response to ^90^Y-transarterial radioembolisation in hepatocellular carcinoma (HCC) [45]. It also has the potential to identify metabolically active tumour remnants after ^90^Y-transarterial radioembolisation in HCC [46].

As CHKα is overexpressed in a number of cancers and has a role in the onset and progression of some cancers, choline kinase inhibitors have been proposed as novel therapeutic targets [47]. CHKα inhibitors are being evaluated in a first in-human phase 1 trial in patients with advanced solid tumours [48]. [^18^F]D4-FCH PET imaging may provide a noninvasive biomarker for developing and assessing the mechanism of action of future choline kinase inhibitors.

## 13. Conclusions

[^18^F]D4-FCH has improved protection against choline oxidase, the key choline catabolic enzyme, via a ^1^H/^2^D isotope effect, together with fluorine substitution. Due to the promising mechanistic and safety profiles of [^18^F]D4-FCH in vitro and preclinically in vivo, the radiotracer has transitioned to clinical development. [^18^F]D4-FCH is a safe PET tracer, with a favourable radiation dosimetry profile for clinical imaging. [^18^F]D4-FCH PET-CT in lung and prostate cancers has shown highly heterogeneous intratumoural distribution and large lesion variability suggesting a use for potential radiotherapy dose escalation and treatment response monitoring.

## Figures and Tables

**Table 1:** Summary of [^18^F]D4-FCH studies: Translation from bench to bedside.

Setting	Findings/Comments
In vitro stability [15], 2011	Chromatographic analyses showed that [^18^F]fluoro-[1,2-^2^H_4_]choline ([^18^F]D4-FCH) was ~80% intact after treatment for 1 h with potassium permanganate, which oxidizes choline to betaine. Conversely, only 40% of [^18^F]Fluorocholine ([^18^F]FCH) was intact. Therefore, [^18^F]D4-FCH is more stable than [^18^F]FCH against oxidation.
In vivo biodistribution [15], 2011	[^18^F]D4-FCH exhibited higher accumulation in the tumours than [^18^F]FCH, particularly at later time points. The distribution of the three radiotracers, [^18^F]FCH, [^18^F]fluoro-[1-^2^H_2_]choline, and [^18^F]D4-FCH, showed a similar uptake profile in most organs, with prominent radioactivity in the kidneys and liver. The plasma concentrations of both tracers were <0.1% ID/g at 2 min after injection, with <10% of [^18^F]FCH intact and ~50% of [^18^F]D4-FCH intact as predicted from the deuterium isotope effect. A reduced uptake of [^18^F]D4-FCH, relative to [^18^F]FCH and [^18^F]fluoro-[1-^2^H_2_]choline, in lung tissue may make imaging of thoracic tumours using this radiotracer superior to [^18^F]FCH.
In vivo response assessment [17], 2009	At day 10 after drug treatment, compared with the pretreatment group, the following observations were seen: Reduction in tumour size by 12%. Marked reduction in radiotracer retention in the treated tumours. Decrease in all imaging variables. [^18^F]D4-FCH can be used for response assessment even under conditions where large changes in tumour size reduction are not seen.
In vitro and in vivo comparison [18], 2012	Deuteration and fluorination combine to provide protection against choline oxidation in vivo. Uptake of [^18^F]D4-choline was the same in three different tumour types, suggesting that [^18^F]D4-choline has utility for cancer detection irrespective of histologic type.
Human biodistribution [19], 2014	[^18^F]D4-FCH was well tolerated, with no radiotracer-related serious adverse events reported. The mean effective dose averaged over both males and females (±SD) was estimated to be 0.025 ± 0.004 (male 0.022 ± 0.002; female 0.027 ± 0.002) mSv/MBq. Highest-absorbed dose (mGy/MBq) was in the kidneys (0.106 ± 0.03), liver (0.094 ± 0.03), pancreas (0.066 ± 0.01), urinary bladder wall (0.047 ± 0.02), and adrenals (0.046 ± 0.01). Elimination was through the renal and hepatic systems. [^18^F]D4-FCH is a safe radiotracer with a dosimetry profile comparable to other common [^18^F]PET tracers.
First in-patient evaluation (lung cancer) [20], 2020	Oxidation of [^18^F]D4-FCH to [^18^F]D4-fluorobetaine was suppressed, confirming the slow catabolism of [^18^F]D4-FCH. Early (5 min) and late (60 min) images showed specific uptake of tracer in all 51 lesions (tumours, lymph nodes, and metastases) from 17 patients analysed. [^18^F]D4-FCH-derived uptake (SUV_max_) in index primary lesions (*n* = 17) ranged between 2.87 and 10.13; lower than that of [^18^F]FDG-PET [6.89, 22.64]. Mathematical modelling demonstrated net irreversible uptake of [^18^F]D4-FCH at steady-state, and parametric mapping of the entire tumour showed large intratumoural heterogeneity in radiotracer retention, which highlights the potential for radiotherapy dose delivery and treatment response monitoring.
Impact of hypoxia on D4-FCH kinetics [21], 2021	The export of phosphorylated [^18^F]D4-FCH and [^18^F]D4-FCHP via HIF-1α-responsive efflux transporters, including ABCB4, when the HIF-1α level is augmented. This is supported by a graphical analysis of human data with a compartmental model (M2T6k + k_5_) that accounts for the efflux. Hypoxia/HIF-1α increases the efflux of phosphorylated radiolabelled choline species, thus supporting the consideration of efflux in the modelling of radiotracer dynamics.
Prostate cancer response assessment (Current Study)	Metastatic castrate-resistant prostate cancer (mCRPC) patients (*n* = 9) prospectively recruited for abiraterone/enzalutamide therapy. [^18^F]D4-FCH was performed at baseline, 4–6 and 12–16 weeks and compared to prostate-specific antigen (PSA), Prostate cancer working group 3 (PCWG3) response criteria and survival duration. Heterogeneity of response: A wide-ranging response profile is seen in both bone and soft-tissue lesions. Changes in [^18^F]D4-FCH PET/CT variables at 12–16 weeks are likely to reflect an escape of individual lesions from selective pressures of therapy. We show bioinformatically, that the choline transporters may also contribute to the cholinic phenotype in addition to the phenotype being a proliferation-independent phenotype in advanced prostate cancer. Strong association between [^18^F]D4-FCH-detectable cholinic phenotype (sum of weighted tumour-to-background ratios (TBRs-wsum)) and PSA response. TBR-wsum could reflect disease burden: Patients with larger TBR-wsum changes survived longer, with a TBR-wsum >30% giving a PFS advantage of >25 months. Superiority of TBR-wsum to PSA change or PCWG3 classification is demonstrated by inability of these latter routine measures to predict survival. TBR-wsum should be explored in future studies (together with individual TBR values signifying heterogeneity) to account for aggregate cholinic phenotype from multiple lesions.

**Table 2:** Patient characteristics of the mCRPC response study.

Pt No.	Age (year)	Metastatic Site (Number)	Drug Used	Baseline Parameters					3-Month Parameters					Other Clinical Parameters			
				PSA (ng/mL)	TBR				PSA (ng/mL)	TBR				PCWG3 (Subsequent Treatment)	Alive/Dead	PFS (m)	OS (m)
					5 min p.i.		30 min p.i.			5 min p.i.		30 min p.i.					
					Sum	Sumw	Sum	Sumw		Sum	Sumw	Sum	Sumw				
1	75	left SV, right iliac bone.	Enzalutamide	95.25	28.45	14.31	38.83	19.42	1.08	3.80	1.98	4.89	1.34	SD (Docetaxel)	Alive	34.63	48.43
2	73	node (1), T8 bone.	Enzalutamide	10.54	19.80	13.98	17.48	10.81	2.22	11.37	5.55	8.76	1.18	PR ^∏^ (Docetaxel)	Dead	11.93	29.17
3	58	bone (sacrum and scapula)	Abiraterone	9.67	13.42	7.87	13.56	7.73	3.02	6.17	3.74	8.91	1.73	SD (Docetaxel)	Alive	47.73	53.80
4	63	multiple bone mets (19), nodes (3)	Abiraterone	33.44	170.51	8.65	191.21	9.52	14.5	181.36	9.60	211.00	0.81	SD (Carboplatin/Etoposide)	Dead	9.13	29.53
5	80	left SV, peritoneal lesions (2), C3 bone (1)	Abiraterone	198	27.91	7.75	23.91	6.45	63.67	30.91	8.29	27.90	1.06	SD (Cabazitaxel)	Dead	14.47	38.67
6	74	bone mets (7), node (1)	Enzalutamide	228.82	18.51	3.34	21.25	3.26	92.95	14.88	2.33	14.88	2.17	SD (Docetaxel)	Dead	12.13	28.10
7	84	right iliac bone (1).	Abiraterone	27.19	2.62	2.62	4.10	4.10	8.17	1.38	1.38	1.69	1.69	SD (^‡^ Nil)	Dead	25.60	25.80
8	74	nodes (2)	Enzalutamide	22.54	7.70	3.50	6.62	3.50	14.90	N/A	N/A	N/A	N/A	SD (Enzalutamide)	Alive	36.00	36.00
9	69	bone (2)	Enzalutamide	94.47	97.32	8.56	98.73	8.77	32.78	N/A	N/A	N/A	N/A	N/A (^†^ Treatment break)	Alive	34.00	36.00

SV, seminal vesicle; TBR, tumour-to-background ratio; p.i., post-injection; PSA, prostate-specific antigen; PCWG3, prostate cancer working group 3 (at 3 months); SD, stable disease; PR, partial response; N/A, not available as overseas. ^∏^ Had nodal disease that responded to treatment on the 3-month follow-up. Subsequently, patient showed multiple (routine PSMA-avid) bone metastases and died from their disease. ^‡^ Nil further due to Dementia. ^†^ Treatment break abroad (in COVID-19 lockdown). Then, progressive disease on return. Radium-223 and radiotherapy.

## Data Availability

Data supporting the conclusions of this manuscript are included within the article and the Supplementary Figures.

## Supplementary Materials

The following supporting information can be downloaded at: https://www.mdpi.com/article/10.3390/molecules28248018/s1, Figure S1: Patient level comparison of PSA with [18F]D4-FCH PET/CT; Figure S2: Patient level prediction of progression-free survival using current approaches and by [18F]D4-FCH PET/CT. Table S1: Methodology of [18F]D4-FCH studies.
