# Supplementary material for: Bench to Bedside Development of [18F]Fluoromethyl-(1,2-2H4)choline ([18F]D4-FCH)
Source: Molecules. 2023 Dec 8;28(24):8018. doi: 10.3390/molecules28248018 (PMC10745874; doi:10.3390/molecules28248018)
Supplement: Supplementary file 1 [file molecules-28-08018-s001.zip › Suppl Table S1.pdf]

Supplementary Table S1: Methodology of [^{18}F]D4-FCH studies

Setting	Methodology
In vitro Stability [15], 2011	Effect of deuterium substitution on bond strength was tested by evaluation of chemical oxidation pattern using potassium permanganate and HPLC
In vivo Biodistribution [15], 2011	In a murine HCT116 human colon xenograft model, ex vivo biodistribution patterns of [^{18}F]fluoro-[1-$^{2}H_2$]choline, [^{18}F]fluoro-[1,2-$^{2}H_4$]choline ([^{18}F]D4-FCH) and [^{18}F]FCH were compared at 2, 30, and 60 min after injection.
In vivo Response assessment [17], 2009	The human colon carcinoma xenograft HCT116 model was treated with mitogenic extracellular kinase inhibitor (PD0325901) daily for 10 days. Dynamic list mode data were acquired after injection of [^{18}F]FCH or [^{18}F]D4-FCH (2.96-3.7 MBq), pre and post treatment. Cumulative images of 30 to 60 min dynamic data were used for visualization of radiotracer uptake and to obtain TACs for each tissue (liver, kidney, muscle, urine, and tumour). Tumour radioactivity was normalized to whole-body radioactivity and expressed as percent injected dose per voxel (%ID/vox). The normalized uptake of radiotracer at 60 min (%ID/vox60) was used for subsequent comparisons. The area under the curve was calculated as the integral of %ID/vox from 0 to 60 min.
Invitro and Invivo comparison [18], 2012	Biodistribution, metabolism, small-animal PET studies, and kinetic analysis of tracer uptake were carried out in human colon (HCT116), melanoma (A375), and prostate cancer (PC3-M) xenograft–bearing mice. Dynamic [^{11}C]choline, [^{11}C]D4-choline, and [^{18}F]D4- choline scans were acquired in list mode format over 60 minutes. Arterial input function was estimated using a single voxel in the centre of the heart cavity using 2 to 5 minutes of cumulative images. Visualization of radiotracer uptake in the tumour; 30- to 60-minute cumulative images of the dynamic data were employed for tumour visualisation and to define ROI.<br>The area under the TAC, calculated as the integral of %ID/mL from 0 to 60 minutes, and the normalized uptake of radiotracer at 60 minutes (%ID/mL60) were also used for comparisons.
Human Biodistribution [19], 2014	[^{18}F]D4-FCH was intravenously administered as a bolus injection (Mean ± SD: 161±2.17; range: 156-163 MBq) to eight healthy volunteers (4-Male; 4-Female). Whole body (vertex to mid-thigh) PET/CT scans were acquired at 6 time points, up to 4 h after tracer injection. Serial whole blood, plasma, and urine samples were collected for radioactivity measurement and plasma radiotracer metabolites. Tissue [^{18}F] radioactivities were determined from quantitative analysis of the images, and TACs were generated. The total numbers of disintegrations in each organ normalized to injected activity (Residence Times) were calculated as the area under the curve of the TAC normalized to injected activities and standard organ volumes. Dosimetry calculations were performed using OLINDA/EXM 1.1.
First in patient evaluation (Lung Cancer) [20], 2020	[^{18}F]D4-FCH (300.5±72.9MBq [147.60-363.6MBq]) was administered intravenously to 17 newly diagnosed NSCLC patients. PET/CT scans were acquired concurrently with radioactive blood sampling to permit mathematical modelling of blood-tissue transcellular rate constants.<br>Comparisons were made with biopsy-derived choline kinase-α (CHKα) expression and diagnostic [^{18}F]FDG scans.
Impact of hypoxia on [18F]D4-FCH kinetics [21], 2021	The effects of hypoxia on [^{18}F]D4-FCH uptake were studied in CHKα-overexpressing cell lines of prostate cancer, PC-3, and breast cancer MDA-MB-231 cells.<br>The mechanisms of radiotracer efflux were assessed by the cell uptake and immunofluorescence in vitro and examined in vivo in a ABCB4-rich PC-9 human non-small cell lung cancer (NSCLC) xenograft model (n = 24).<br>The mathematical modelling methodology was further developed to verify the efflux hypothesis using [^{18}F]D4-FCH dynamic PET scans from NSCLC patients.

Prostate cancer response assessment (current study)	Nine mCRPC patients (mean age 72.5 years, range 58-84) were prospectively recruited pre abiraterone/enzalutamide therapy between May 2017 and Nov 2018. [18F]D4-FCH was performed at baseline, 4-6 and 12-16 weeks and compared to PSA, PCWG3 response criteria and survival duration. PET images were analysed by a single experienced observer. PET positive tumour index lesions ≥ 10 mm (focal areas of increased tracer uptake above normal physiological distribution) were manually outlined to generate VOIs from which lesion SUV_{max} and SUV_{mean} were documented. Normal background organs were outlined to generate background SUV_{mean} (2 cm fixed sphere iliac crest bone not involved by tumour; 3 cm fixed sphere gluteal muscle; 3 cm fixed sphere liver; 2 cm fixed sphere mediastinal blood pool at level of main pulmonary artery). . For all index lesions, TBRs were documented (SUV_{max} lesion/ SUV_{mean} background) using background muscle for nodes and soft-tissue lesions and background bone for bone metastases.In all cases, the VOIs from the 5 minute acquisition were copied to the 30 and 60 minute time points and manually adapted if any patient movement had occurred between scans. For lesions no longer visible above background, the background activity at the site of the lesion was outlined.<br>The patients' standard imaging CECT and bone scans were evaluated by one experienced observer blinded to the PET data and response was evaluated according to PCWG-3 guidelines.<br>Per patient analyses were performed assessing the mean % change of each parameter (SUV_{max} , SUV_{mean} and TBR) using a) all lesions b) maximum of 5 lesions per patients (2 per organ) 24. To obtain a measure of uptake in all lesions within a patient, we calculated the TBRwsum variable as the weighted sum of the TBR values in all lesions according to the equation:<br>TBRwsum = Σi (wi*TBRi)/Σi wi with i=[1,N] with N=number of lesions,<br>where wi is the weight relative to the i-th lesion (with a TBR = TBRi). Weights will be proportional to the uptake of the lesion (e.g., w = 1 for the hottest lesion).<br><br>The median (range) was used as the primary descriptive measure. The association between baseline PET parameters and PSA, and between changes in PET parameters and PSA, was assessed using Pearson's correlation test. PFS was defined as the time from the date of start of abiraterone or enzalutamide and the date of progression (start of subsequent treatment) or the date of last follow up. OS was defined as the time from the date of start of abiraterone or enzalutamide and the date of death or last follow up. Analysis of PFS and OS was performed by Kaplan-Meier estimates and log rank test. P values ≤ 0.05 were considered significant. All statistical tests were run in Matlab (Mathworks, R2018b).

CECT, contrast enhanced computed tomography; FDG, [18F]fluorodeoxyglucose; HPLC, high performance liquid chromatography; metastatic castration-resistant prostate cancer mCRPC; PSA, prostate specific antigen; PCWG3, prostate cancer working group 3; PFS, progression free survival; NSCLC, non small cell lung cancer; OS, overall survival; ROI, regions of interest; SD, standard deviation; SUV, standardised uptake value; TACS, time versus radioactivity curves; TBR, tumour to background ratio; VOI, volumes of interest
