# Supplementary material for: Bench to Bedside Development of [18F]Fluoromethyl-(1,2-2H4)choline ([18F]D4-FCH)
Source: Molecules. 2023 Dec 8;28(24):8018. doi: 10.3390/molecules28248018 (PMC10745874; doi:10.3390/molecules28248018)
Supplement: Supplementary file 1 [file molecules-28-08018-s001.zip › SUPPLEMENTARY FIGURES.pdf]

SUPPLEMENTARY FIGURES

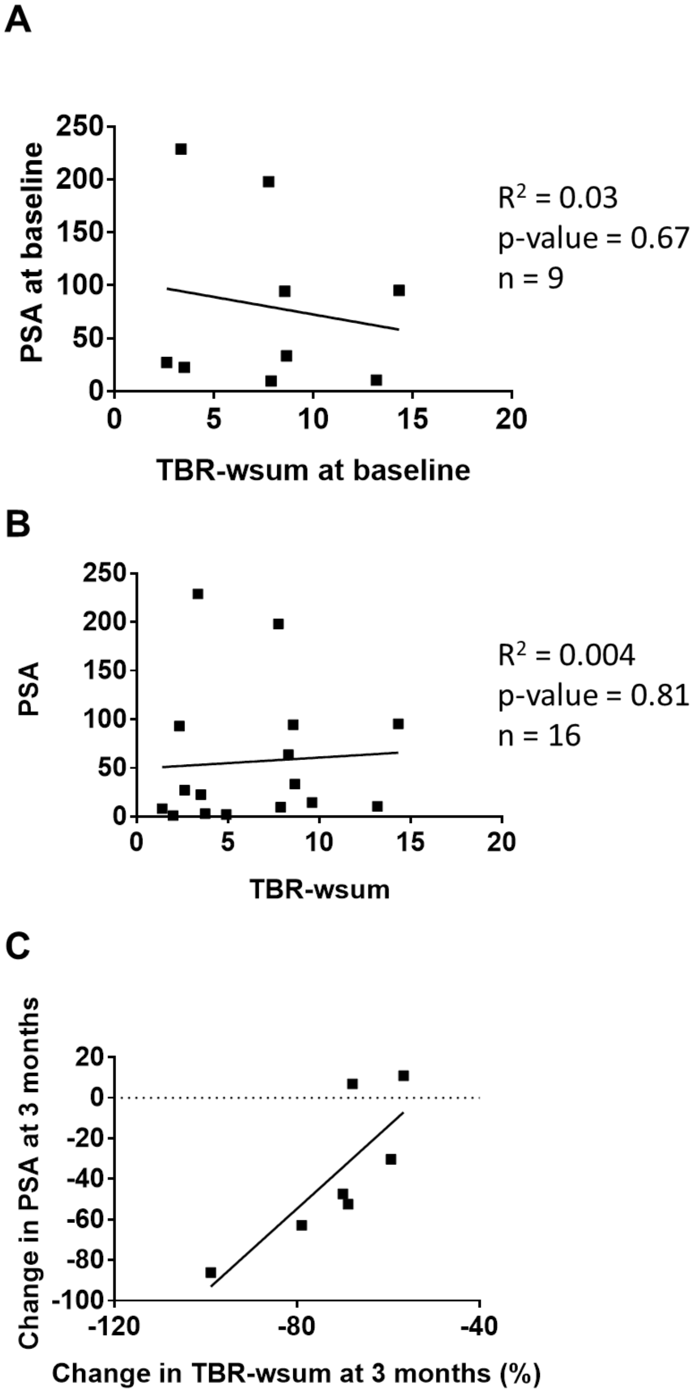

Supplementary Figure S1. Patient level comparison of PSA with [^{18}F]D4-FCH PET/CT. A) Baseline PSA and [^{18}F]D4-FCH PET/CT derived TBR-wsum, B) PSA at any time point and TBR-wsum, and C) Change in PSA and change in TBR-wsum determined at 3 months relative to baseline.

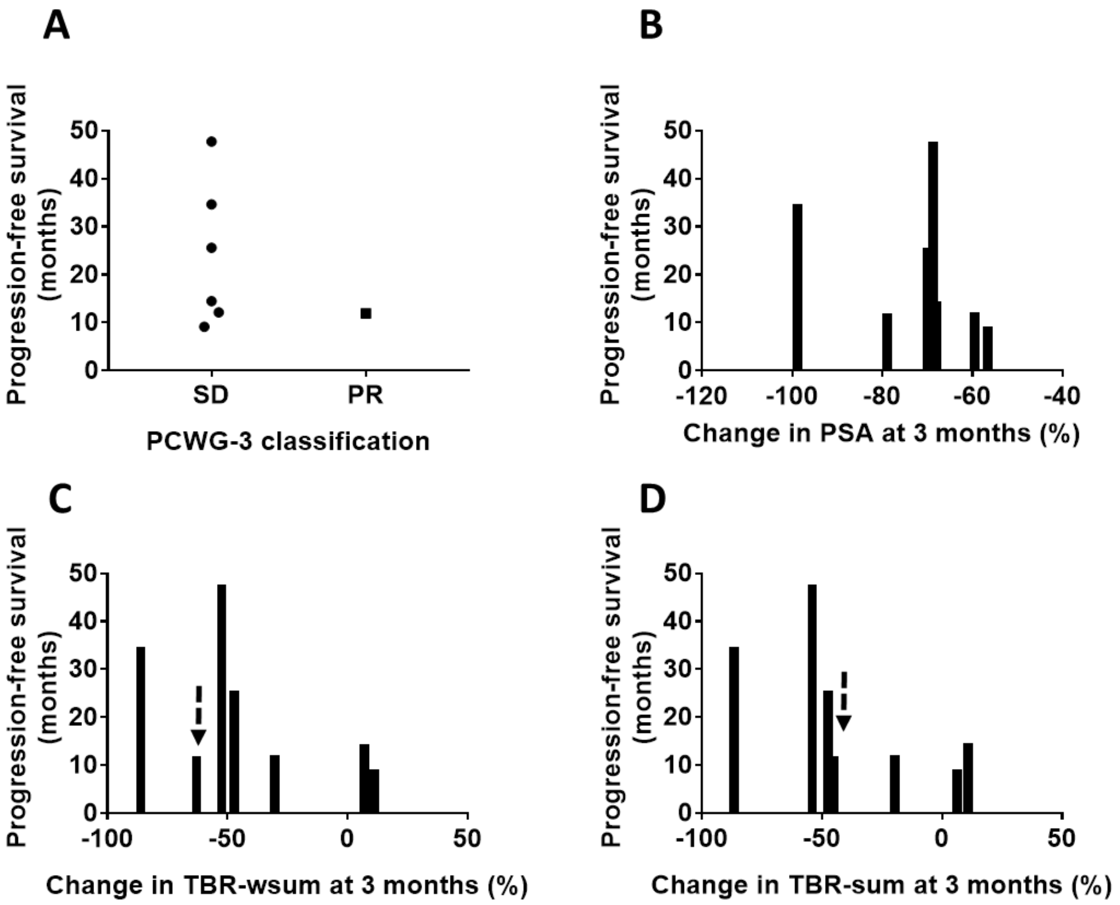

Supplementary Figure S2. Patient level prediction of progression-free survival using current approaches and by [18F]D4-FCH PET/CT. A) PCWG-3 classification and progression-free survival. B) PSA change at 3 months and progression-free survival. C) [18F]D4-FCH PET/CT TBR-wsum change at 3 months and progression-free survival. Arrow represents patient #2 who had discordant conventional versus PET scoring. D) Change in [18F]D4-FCH PET/CT TBR-sum - representing sum of all lesions – rather than a weighted change. Arrow represents patient #2 who had discordant conventional versus PET scoring.
